# Maternal high-fat diet orchestrates offspring hepatic cholesterol metabolism via MEF2A hypermethylation-mediated CYP7A1 suppression

**DOI:** 10.1186/s11658-024-00673-8

**Published:** 2024-12-18

**Authors:** Ling Zhang, Wenyu Zou, Shixuan Zhang, Honghua Wu, Ying Gao, Junqing Zhang, Jia Zheng

**Affiliations:** Department of Endocrinology, Peking University First Hospital, No. 8 Xishiku Ave, Xicheng Beijing, 100034 People’s Republic of China

**Keywords:** maternal HFD, hepatic cholesterol metabolism, MEF2A, CYP7A1, DNA methylation, offspring

## Abstract

**Background:**

Maternal overnutrition, prevalent among women of childbearing age, significantly impacts offspring health throughout their lifetime. While DNA methylation of metabolic-related genes mediates the transmission of detrimental effects from maternal high-fat diet (HFD), its role in programming hepatic cholesterol metabolism in offspring, particularly during weaning, remains elusive.

**Methods:**

Female C57BL/6 J mice were administered a HFD or control diet, before and during, gestation and lactation. Hepatic cholesterol metabolism genes in the liver of offspring were evaluated in terms of their expression. The potential regulator of cholesterol metabolism in the offspring’s liver was identified, and the function of the targeted transcription factor was evaluated through in vitro experiments. The methylation level of the target transcription factor was assessed using the MassARRAY EpiTYPER platform. To determine whether transcription factor expression is influenced by DNA methylation, in vitro experiments were performed using 5-azacitidine and Lucia luciferase activity assays.

**Results:**

Here, we demonstrate that maternal HFD results in higher body weight and hypercholesterolemia in the offspring as early as weaning age. Maternal HFD feeding exacerbates hepatic cholesterol accumulation in offspring primarily by inhibiting cholesterol elimination to bile acids, with a significant decrease of hepatic cholesterol 7α-hydroxylase (CYP7A1). RNA-seq analysis identified myocyte enhancer factor 2A (MEF2A) as a key transcription factor in the offspring liver, which was significantly downregulated in offspring of HFD-fed dams. MEF2A knockdown led to CYP7A1 downregulation and lipid accumulation in HepG2 cells, while MEF2A overexpression reversed this effect. Dual luciferase reporter assays confirmed direct modulation of CYP7A1 transcription by MEF2A. Furthermore, the reduced MEF2A expression was attributed to DNA hypermethylation in the *Mef2a* promoter region. This epigenetic modification manifested as early as the fetal stage.

**Conclusions:**

This study provides novel insights into how maternal HFD orchestrates hepatic cholesterol metabolism via MEF2A hypermethylation-mediated CYP7A1 suppression in offspring at weaning.

## Introduction

In recent decades, obesity has become more prevalent among women of childbearing age, becoming an escalating issue worldwide [1, 2]. Maternal overnutrition predisposes offspring to risks of obesity and diabetes, persisting throughout their lifetime [3, 4]. Metabolic abnormalities in mothers, including hyperglycemia, hyperlipidemia, insulin resistance, oxidative stress, and chronic inflammation, create an adverse intrauterine environment. This environment influences fetal growth and development, ultimately reprogramming offspring metabolic health over generations [5, 6]. Substantial evidence, including our previous studies, has revealed that offspring exposed to a maternal high-fat diet (HFD) exhibit higher body weight, glucose intolerance, and dyslipidemia at weaning age and in adulthood [7–12]. Nevertheless, the precise mechanism underlying this transgenerational phenomenon remains uncertain.

DNA methylation plays a pivotal role in regulating key metabolic genes through epigenetic mechanisms, recognized as a significant factor in the intergenerational transmission of metabolic disorders [13–15]. Studies have shown alterations in DNA methylation patterns of peroxisome proliferator-activated receptor-α (PPARα) and leptin in the oocytes of HFD-fed dams, as well as in the offspring’s livers [14]. Du et al. found that maternal HFD has been linked to increased DNA methylation in Dio3 antisense RNA (*Dio3os*) in fetal brown adipose tissue, persisting into adulthood and contributing to obesity and metabolic dysfunctions in offspring [15]. These findings underscore the role of DNA methylation of key genes as an epigenetic mechanism underlying the adverse effects of maternal HFD consumption on metabolic health in offspring.

It is crucial to maintain cholesterol homeostasis for proper cellular and systemic functions. Cholesterol levels are regulated by several biological processes, including cholesterol uptake [low-density lipoprotein receptor (LDLR) and scavenger receptor class B type I (SR-BI)], cholesterol biosynthesis [3 hydroxy-3 methylglutaryl CoA reductase (HMGCR)], cholesterol degradation [cholesterol 7α-hydroxylase (CYP7A1) and cytochrome P450 family 8 subfamily B member 1 (CYP8B1)], and cholesterol efflux [ATP binding cassette subfamily G member 5 (ABCG5) and ATP binding cassette subfamily G member 8 (ABCG8)] [16]. Notably, hepatic CYP7A1 serves as a rate-limiting enzyme for converting cholesterol to bile acids in the degradation process [17]. The disturbance of cholesterol homeostasis is critical to the development of multiple diseases, including nonalcoholic fatty liver disease and cardiovascular disease, neurodegenerative diseases, and cancer [16, 18, 19]. Thus, attention to cholesterol metabolism from early life is necessary. A growing body of epidemiological and experimental evidence suggests that both hypercholesterolemia and nonalcoholic fatty liver disease are intrauterine development-related diseases [20, 21]. Adverse conditions during pregnancy can directly influence the offspring’s liver cholesterol metabolism both in utero and for years after birth [21]. Human offspring with maternal metabolic disorders, including gestational diabetes mellitus and obesity, have increased liver fat content [22]. These studies indicate the importance of adverse maternal environments on cholesterol metabolism disorders in offspring.

However, studies on the programming effects of maternal HFD on hepatic cholesterol metabolic function in offspring are predominantly descriptive, and the mechanisms responsible for these effects have not yet been well elaborated. Therefore, we aim to investigate how maternal HFD affects offspring’s hepatic cholesterol metabolism, particularly during weaning age. Additionally, we will explore the potential role of DNA methylation of key genes in programming these transgenerational effects.

## Materials and methods

### Animals and experimental design

Five-week-old female C57BL/6 J mice were obtained and maintained under standard conditions at 22 ± 2 °C with a 12-h light/12-h dark cycle. Following 1-week acclimatization, 6-week-old female mice were randomly fed with a standard diet (13% fat, 24% protein, 63% carbohydrate, 3.44 kcal/g) (F0-Ctrl for dams) or a HFD (60% fat, 20% protein, 20% carbohydrate, 5.24 kcal/g) (F0-HFD for dams) for a duration of 4 weeks. Subsequently, 10-week-old female mice were mated with standard-diet-fed male mice (female:male = 2:1) to control for potential differences in sires. Observation of vaginal plugs was designated embryonic day 0.5 (E0.5). Throughout gestation and until pup weaning, females were maintained on their respective diets. Two cohorts of dams were established for this study. In the first cohort, dams were anesthetized on gestational day 18.5, and fetal livers were collected. Experiments were conducted on the fetus irrespective of the sex of the pups. In the second cohort, each dam had a litter size of 6–10, and the litter size was standardized to six mice per litter post birth to avoid nutritional bias, irrespective of the sex of the pups. At weaning, we randomly selected one male offspring from each litter for further analysis (F1-HFD and F1-Ctrl for offspring, respectively). A 12-h fast was followed by anesthesia and sacrifice, and blood and livers were immediately harvested and stored at −80 °C. The weights of subcutaneous and visceral adipose tissue were recorded.

### Measurement of lipids concentrations

Serum total cholesterol (T-CHO), low-density lipoprotein cholesterol (LDL-C), and triglyceride (TG) were quantified using commercial kits (A111-1, A113-1, A110-1, respectively, Jiancheng Bioengineering Institute, Nanjing, China). Hepatic T-CHO and TG contents were extracted from liver samples and quantitated using the same commercial kits (A111-1, A110-1, respectively; Jiancheng Bioengineering Institute, Nanjing, China). Each sample was assayed in duplicate.

### Oil Red O staining

Liver samples were embedded in Tissue-Tek O.C.T. compound and sectioned at 10 µm thickness for each mouse. Oil Red O staining was performed by immersing slides in 60% isopropanol for 2 min, followed by staining with working Oil Red O for 10 min (G1015, ServiceBio, Beijing, China). After brief rinsing with distilled water and 60% isopropanol, slides were stained with hematoxylin (G1004, ServiceBio, Beijing, China) for 15 s. Images were captured using an Olympus DP71 microscope.

### Hematoxylin–eosin (H&E) staining and immunohistochemistry

For H&E staining, liver sections (5 μm) were sequentially incubated in hematoxylin for 15 s and eosin for 30 s (G1002, ServiceBio, Beijing, China). For immunohistochemistry, liver sections were pretreated in citrate buffer (PH 6.0, 98 °C) for 20 min, followed by quenching of endogenous peroxidase activity with 0.3% H_2_O_2_ for 15 min. After blocking in goat serum (C0265, Beyotime, Beijing, China) for 20 min, sections were incubated with primary antibodies against myocyte enhancer factor 2A (MEF2A) (1:1000, ab264329, Abcam, Cambridge, UK) and CYP7A1 (1:1000, 18054–1-AP, Proteintech, Wuhan, China) overnight at 4 °C. The next day, sections were incubated with a secondary antibody (PV9000, Zhongshan Gold Bridge Biotechnology Co, Beijing, China) for 1 h at room temperature. Visualization was achieved using diaminobenzidine (DAB) staining (ZLI9018, Zhongshan Gold Bridge Biotechnology Co, Beijing, China), followed by counterstaining with hematoxylin. Images were captured with an Olympus DP71 microscope. Five random fields were selected from each section, and mean integrated optical density (IOD/area) was quantified using Image-Pro Plus 6.0 software.

### Quantitative real-time PCR (qRT-PCR)

Total RNA was extracted from liver tissues or cells using TRIzol reagent (15596026, Invitrogen, Waltham, MA, USA), and 1 µg of RNA was reverse transcribed into cDNA using the high-capacity cDNA reverse transcription kit (4375222, Thermo Fisher Scientific, Hudson, NH, USA). Gene expression was analyzed using SYBR Green (A25742, Thermo Fisher Scientific, Hudson, NH, USA). β-Actin was used as an endogenous control. Primer sequences are listed in Table 1.

**Table 1:** Primer sequences of genes for quantitative RT-PCR analysis

Genes	Forward 5′-3′	Reverse 5′-3′
Mouse		
* Ldlr*	TGACTCAGACGAACAAGGCTG	ATCTAGGCAATCTCGGTCTCC
* Sr-bi*	TTTGGAGTGGTAGTAAAAAGGGC	TGACATCAGGGACTCAGAGTAG
* Hmgcr*	CTTGTGGAATGCCTTGTGATTG	AGCCGAAGCAGCACATGAT
* Cyp7a1*	AAACTCCCTGTCATACCACAAAG	TTTCCATCACTTGGGTCTATGC
* Cyp8b1*	CCTCTGGACAAGGGTTTTGTG	GCACCGTGAAGACATCCCC
* Abcg5*	AGGGCCTCACATCAACAGAG	GCTGACGCTGTAGGACACAT
* Abcg8*	CTGTGGAATGGGACTGTACTTC	GTTGGACTGACCACTGTAGGT
* Dnmt1*	AAGAATGGTGTTGTCTACCGAC	CATCCAGGTTGCTCCCCTTG
* Dnmt3a*	GATGAGCCTGAGTATGAGGATGG	CAAGACACAATTCGGCCTGG
* Dnmt3b*	AGCGGGTATGAGGAGTGCAT	GGGAGCATCCTTCGTGTCTG
* Tet1*	ACACAGTGGTGCTAATGCAG	AGCATGAACGGGAGAATCGG
* Tet2*	AGAGAAGACAATCGAGAAGTCGG	CCTTCCGTACTCCCAAACTCAT
* Tet3*	TGCGATTGTGTCGAACAAATAGT	TCCATACCGATCCTCCATGAG
* Mef2a*	CAGGTGGTGGCAGTCTTGG	TGCTTATCCTTTGGGCATTCAA
* Esr1*	CCTCCCGCCTTCTACAGGT	CACACGGCACAGTAGCGAG
* Bach1*	TGAGTGAGAGTGCGGTATTTGC	GTCAGTCTGGCCTACGATTCT
* Cebpz*	AGAAAGGCTACTCTTCCGCTC	GGGAGAGGACCATTTGGTTTAGA
* Jund*	GGCGGGATTGAAACCAGGG	AGCCCGTTGGACTGGATGA
* Esrra*	CTCAGCTCTCTACCCAAACGC	CCGCTTGGTGATCTCACACTC
* Fosl2*	CCAGCAGAAGTTCCGGGTAG	GTAGGGATGTGAGCGTGGATA
* Yy1*	CAGTGGTTGAAGAGCAGATCAT	AGGGAGTTTCTTGCCTGTCAT
* Hnf4α*	CACGCGGAGGTCAAGCTAC	CCCAGAGATGGGAGAGGTGAT
* β-actin*	TATTGGCAACGAGCGGTTCC	GGCATAGAGGTCTTTACGGATGTC
Human		
* Mef2a*	GGTCTGCCACCTCAGAACTTT	CCCTGGGTTAGTGTAGGACAA
* Cyp7a1*	GCAATTTGGTGCCAATCCTCT	GCACAACACCTTATGGTATGACA
* β-actin*	CATGTACGTTGCTATCCAGGC	CTCCTTAATGTCACGCACGAT

*Ldlr* low-density lipoprotein receptor, *Sr-bi* scavenger receptor class B type I, *Hmgcr* 3-hydroxy-3 methylglutaryl CoA reductase, *Cyp7a1* cholesterol 7α-hydroxylase, *Cyp8b1* cytochrome P450 family 8 subfamily B member 1, *Abcg5* ATP binding cassette subfamily G member 5, *Abcg8* ATP binding cassette subfamily G member 8, *Dnmt* DNA methyltransferases, *Tet* ten-eleven translocation dioxygenases, *Mef2a* myocyte enhancer factor 2A, *Esr1* estrogen receptor 1, *Bach1* BTB domain and CNC homolog 1, *Cebpz* CCAAT enhancer binding protein zeta, *Jund* JunD proto-oncogene, *Esrra* estrogen-related receptor alpha, *Fosl2* Fos like 2, AP-1 transcription factor subunit, *Yy1* Yy1 transcription factor, *Hnf4α* hepatocyte nuclear factor 4α, *Ap-1* activator protein-1

### Western blots

Hepatic tissues and cells were lysed in RIPA lysis buffer (PLB004N-BR100, Snodetech, Beijing, China). Total protein was separated by 10% SDS-PAGE gel and transferred onto nitrocellulose membranes. After blocking for 1 h in 5% fat-free milk, membranes were incubated overnight at 4 °C with primary antibodies against DNA methyltransferases 1 (DNMT1) (1:1000, #5032, Cell Signaling Technology, Danvers, MA, USA), DNMT3A (1:1000, 20,954–1-AP, Proteintech, Wuhan, China), DNMT3B (1:1000, 26,971–1-AP, Proteintech, Wuhan, China), MEF2A (1:1000, Ab76063, Abcam, Cambridge, UK), CYP7A1 (1:1000, 18,054–1-AP, Proteintech, Wuhan, China), and β-actin (1:1000, #4970 s, Cell Signaling Technology, Danvers, MA, USA). After washing, membranes were incubated with a secondary antibody (1:5000, Zhongshan Gold Bridge Biotechnology Co, Beijing, China) for 1 h at room temperature. Protein bands were visualized using an enhanced chemiluminescence (ECL) detection kit. β-Actin was used as an endogenous control.

### RNA sequencing (RNA-seq) analyses

RNA was quantified using the ND-2000 (NanoDrop Technologies). To construct sequencing libraries, the OD260/280 ratio (1.8 × 2.2), the OD260/230 ratio (2.5 × 2.0), the RIN, and the 28S:18S ratio (1.0 × 1.0) of the RNA samples (> 1ug) were tested. Following the manufacturer’s instructions (Illumina, San Diego, CA), RNA was purified, reverse transcribed, library constructed, and sequenced at Shanghai Majorbio Biopharm Biotechnology Co., Ltd.. A HISAT2 alignment was performed on clean reads to determine their alignment to the reference genome [23]. Each sample’s mapped reads were assembled using StringTie using reference data [24]. Differential expression analysis was performed using the DESeq2, which is a software package for estimating variance−mean dependence and testing for differential expression based on a negative binomial model [25]. Differentially expressed genes (DEGs) with |log_2_FC|> 0 and *P* < 0.05 were considered to be significant, as in previous studies [26, 27]. Functional-enrichment analyses including Gene Ontology (GO) and Kyoto Encyclopedia of Genes and Genomes (KEGG) were performed to identify which DEGs were significantly enriched in GO terms and metabolic pathways, at Bonferroni-corrected *P* value < 0.05, compared with the whole-transcriptome background. GO functional enrichment and KEGG pathway analysis were carried out by Goatools and Python SciPy software, respectively. Predicting possible upstream transcription factors of DEGs was performed using bioinformatics databases, including Animal Transcription Factor Database (AnimalTFDB, http://bioinfo.life.hust.edu.cn/AnimalTFDB4/) and ChIP-X Enrichment Analysis 3 (ChEA3, https://maayanlab.cloud/chea3/).

### Methylation analysis

Hepatic genomic DNA was extracted using the E.Z.N.A. DNA kit (Omega Bio-tek, Inc., Norcross, GA). Promoter methylation of the target gene was detected using the MassARRAY EpiTYPER platform (Agena Bioscience, San Diego, CA, USA). The target region of *Mef2a* was amplified with specific primers. The forward sequence of the primer was 5′-aggaagagagGTTGTGGTATAAGGATAAGAGGGG-3′ and reverse sequence of the primer was 3′-cagtaatacgactcactatagggagaaggctTAACCCAACAACTATCCAAACCTAA-5′. DNA methylation analysis was performed using MassARRAY EpiTYPER software (Agena Bioscience, San Diego, CA, USA).

### Cell culture

Human hepatocellular carcinoma cell lines (HepG2) (SCSP-510) and H9C2 cells (SCSP-5211) were obtained from the Chinese National Infrastructure of Cell Line Resource. HepG2 cells were cultured in MEM medium (11090081, Invitrogen, Waltham, MA, USA) supplemented with 10% fetal bovine serum (FBS), 1% nonessential amino acids (11140050, Invitrogen, Waltham, MA, USA), 1% glutamax (35050061, Invitrogen, Waltham, MA, USA), 1% sodium pyruvate 100 mM solution (11360070, Invitrogen, Waltham, MA, USA), and 1% penicillin–streptomycin (15140122, Gibco, Life Science, Pittsburgh, PA, USA), and maintained at 37 °C with 5% CO_2_. H9C2 cells were cultured in DMEM medium (10566016, Gibco, Life Science, Pittsburgh, PA, USA) supplemented with 10% FBS and 1% penicillin–streptomycin. For LDL-C treatment, HepG2 cells were incubated with 200 mg/dl LDL-C for 24 h (YB-001, Yiyuan biotech, Guangzhou, China). For 5-azacitidine (5-AZA) treatment, HepG2 cells were incubated with 0 mmol/L to 1 mmol/L 5-AZA for 24–48 h (A2385, Sigma, Steinheim, Germany).

### Transient knockdown experiment

Human MEF2A siRNA and scrambled siRNA were designed and synthesized by GenePharma (Shanghai, China). The sequences of siRNA targeting *Mef2a* were used as GGGCAGUUAUCUCAGGGUUTT (sense sequence) and AACCCUGAGAUAACUGCCCTT (antisense sequence), and the sequences of scramble siRNA were UUCUCCGAACGUGUCACGUdTdT (sense sequence), and ACGUGACACGUUCGGAGAAdTdT (antisense sequence). Quantities of 50 nM of each siRNA duplexes were transfected into cells growing in 12-well plates using Lipofectamine RNAiMAX (13,778–030, Invitrogen) for 24 h according to the manufacturer’s instructions. The silencing efficiency was verified by qRT-PCR and western blot, with scrambled siRNA used as a negative control.

### Generation of stable MEF2A overexpression HepG2 cell line by lentivirus

A stable HepG2 cell line overexpressing MEF2A was established using a lentiviral-based delivery system. MEF2A and control plasmids were synthesized by Ubigene (Guangzhou, China). HEK293T cells were seeded in a 10-cm culture dish. Lentiviral and packaging vectors (pCMV-VSVG:psPAX2:target plasmid = 5 μg:15 μg:10 μg) were transfected into the cells on the following day. After 48 h, the cells were harvested and filtered through a 0.45-µm membrane. Lentiviral particles were supplemented with 5 μg/mL polybrene. Afterward, lentiviral particles were added to HepG2 cells and incubated for 24 h. The cells were then supplemented with fresh medium and further incubated for 48 h. Stable cells were selected using puromycin (1 µg/mL) for the following experiments.

### Lucia luciferase activity assay for DNA methylation

To investigate the regulatory effect of CpG methylation in the *Mef2a* promoter region in vitro, a human promoter containing 2000 bp of *Mef2a* was inserted into a reporter plasmid CpG-free basic Lucia (pcpgf-baslc, InvivoGen, San Diego, CA, USA), which lacks CpG sites. The recombinant *Mef2a* plasmid was methylated using M.SssI (M0226S, NEB) according to the manufacturer’s instructions, and then purified and recovered using the Monarch PCR and DNA Cleanup Kit (T1030S, NEB). H9C2 cells were transfected with 200 ng of unmethylated or methylated pCpG free-basic-Lucia plasmid along with 10 ng of firefly luciferase reporter vector. Lucia luciferase activity was measured with QUANTI-Luc (rep-qlc4lg1, InvivoGen, San Diego, CA, USA). The firefly luciferase activity was measured using the Bio-Lite Luciferase Assay System (DD1201-01, Vazyme, Nanjing, China). Lucia luciferase activity was normalized against firefly luciferase activity.

### Dual luciferase reporter assay

The *Cyp7a1* promoter-luciferase reporter construct containing 2.0 kb of the H_*Cyp7a1* promoter sequence (-1950 to +50) and the pRL *Renilla* luciferase control reporter vector was constructed by Genomeditech (Guangzhou, China). HepG2 cells were seeded in 12-well plates, and then transfected with 200 ng of *Cyp7a1*-luciferase reporter and 20 ng pRL *Renilla* along with 200 ng of *Mef2a* plasmid. Luciferase activity was determined 48 h post-transfection using the Dual-Luciferase Reporter Assay System (E1910, Promega Corp, WI, USA). Relative luciferase activity was normalized against *Renilla* luciferase activity.

### Statistical analysis

Statistical analyses were performed using GraphPad Prism 10.0 software. Data are presented as mean ± standard error (SEM). Student’s unpaired *t*-test was used for comparisons between the two groups, while one-way analysis of variance (ANOVA) followed by Tukey’s post hoc test was utilized for multiple comparison analysis. *P* value < 0.05 was considered statistically significant.

## Results

### HFD impairs lipid metabolism in dams

**Fig. 1:**
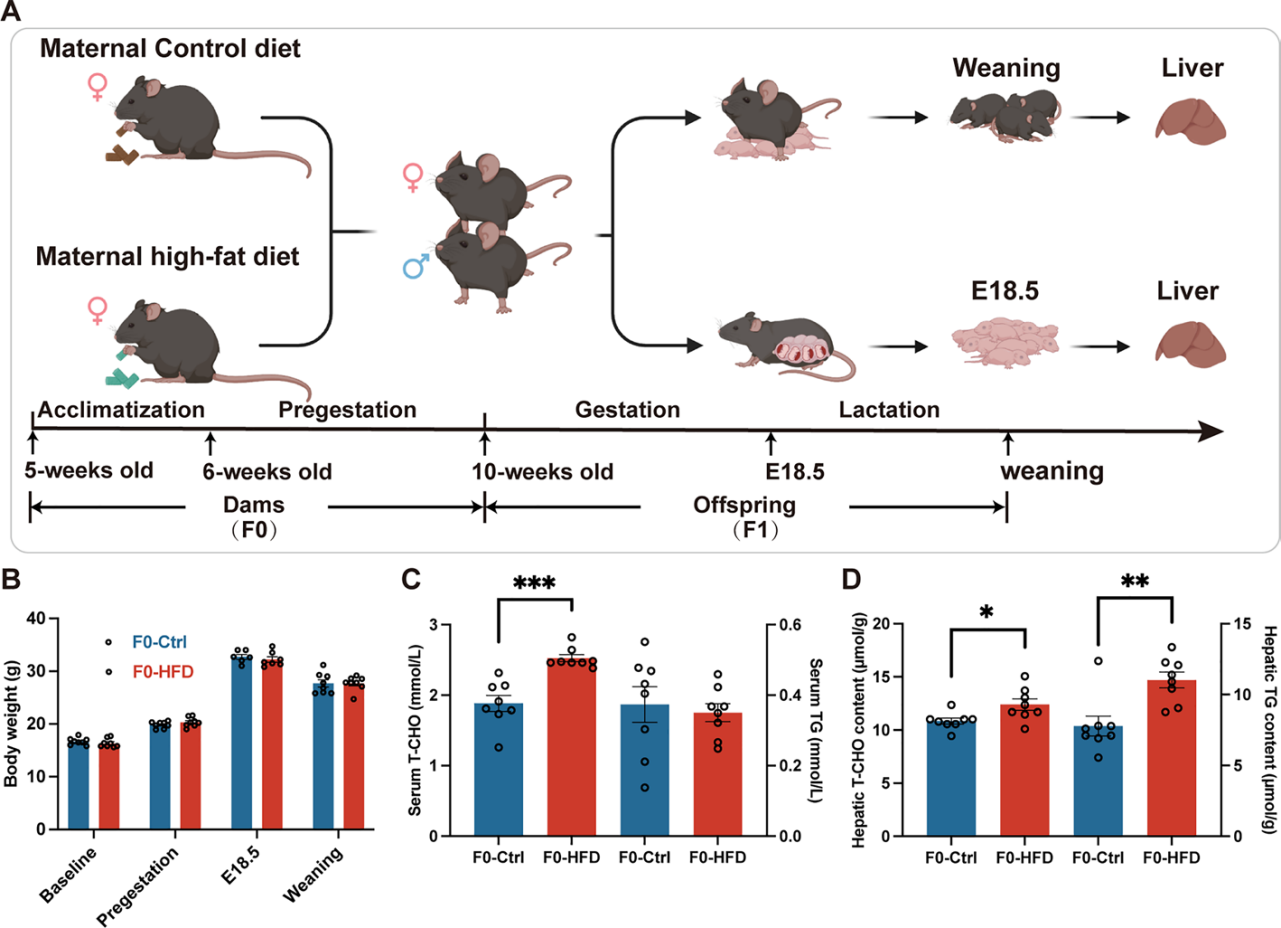
Maternal high-fat diet impairs lipid metabolism in dams. **A** Scheme of experimental design. **B** Body weight of dams prior to or during gestation (E18.5) and lactation (at weaning). **C** Serum T-CHO and TG concentrations in dams. **D** Hepatic T-CHO and TG contents in dams. F0, the first generation (dam); Ctrl, standard diet; HFD, high-fat diet; T-CHO, total cholesterol; TG, triacylglycerol. Data presented as the mean ± SEM. *P < 0.05, **P < 0.01, ***P < 0.001 versus F0-Ctrl group. *n* = 6–8 litters for F0-Ctrl group, *n* = 7–8 litters for F0-HFD group

The scheme of the experimental design is shown in Fig. 1A). The body weight of each dam was regularly monitored, and no significant difference in body weight was observed between dams fed with a standard diet and a high-fat diet prior to breeding, during gestation, and lactation (Fig. 1B). However, maternal HFD exposure increased serum total cholesterol (T-CHO) level, without affecting serum triglyceride (TG) concentration in offspring at weaning (Fig. 1C). Moreover, both hepatic T-CHO and TG contents were elevated in dams of the HFD group, compared with controls (Fig. 1D). These results suggest that dams fed with HFD displays higher plasma cholesterol and impairs lipid metabolism.

### Maternal HFD disturbs hepatic lipid metabolism in offspring at weaning

**Fig. 2:**
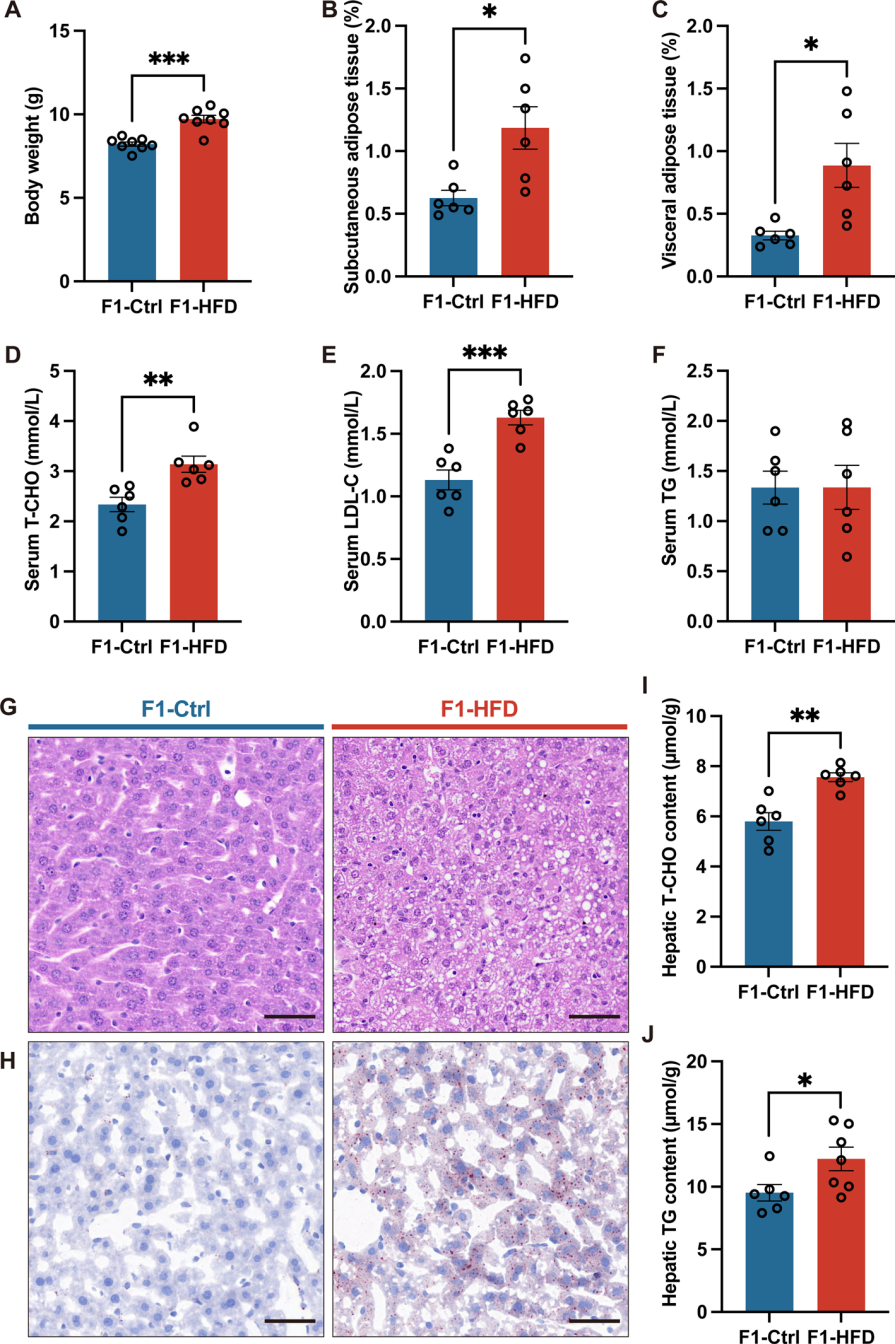
Maternal high-fat diet induces lipid metabolism disturbances in offspring at weaning. **A** Body weight of offspring at weaning. **B** Subcutaneous adipose tissue (%). **C** Visceral adipose tissue (%). **D** Serum T-CHO concentration. **E** Serum LDL-C concentration. **F** Serum TG concentration. **G** Representative images of H&E staining of the liver. **H** Representative images of Oil Red O staining of the liver. **I** Hepatic T-CHO content. **J** Hepatic TG content. The scale bar indicates 50 μm. F1, the second generation (offspring); Ctrl, standard diet; HFD, high-fat diet; T-CHO, total cholesterol; LDL-C, low-density lipoprotein cholesterol; TG, triacylglycerol. Data presented as the mean ± SEM. *P < 0.05, **P < 0.01, ***P < 0.001 versus F1-Ctrl group. *n* = 6–8 litters for F1-Ctrl group, *n* = 6–8 litters for F1-HFD group. one male offspring per litter

Further measurements were made at the weaning of male offspring to determine metabolic parameters from the previous two dam cohorts. Male offspring from HFD dams exhibited increased body weight at weaning compared with those from control diet (Ctrl) dams (Fig. 2A). Additionally, offspring from HFD-fed dams showed increased subcutaneous and visceral adipose tissue contents (% body weight) (Fig. 2B, C). A higher level of TCHO and LDL-C was found in the serum of offspring whose dams consumed HFD compared with the control group, whereas serum TG levels showed no differences (Fig. 2D–F).

We further evaluated hepatic lipid deposition in offspring mice. Offspring from HFD-fed dams exhibited a massive accumulation of lipid droplets in the liver, as confirmed by H&E staining and Oil Red O staining (Fig. 2G, H). Consistently, hepatic T-CHO and TG contents were higher in offspring from HFD-fed dams compared with those from control diet dams (Fig. 2I, J). The findings suggest that maternal HFD feeding disrupts hepatic lipid metabolism at weaning in offspring.

### Maternal HFD consumption impairs hepatic cholesterol metabolism by suppressing CYP7A1 expression in offspring at weaning

**Fig. 3:**
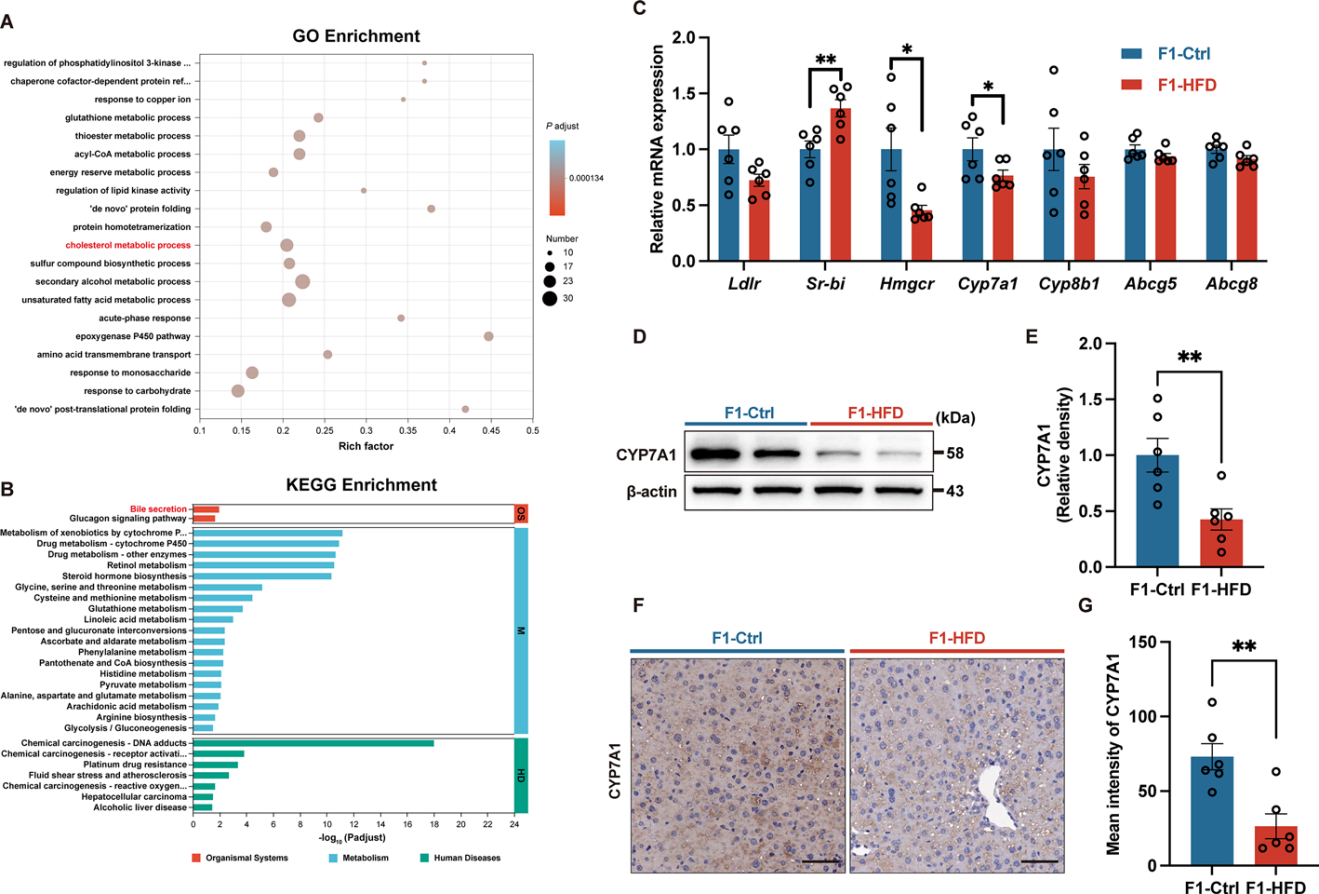
Maternal high-fat diet disturbs hepatic cholesterol metabolism by suppressing CYP7A1 expression in offspring at weaning. **A** GO enrichment analysis. **B** KEGG pathway analysis. **C** mRNA expression of genes involved in cholesterol metabolism. **D** Representative immunoblot images for CYP7A1. **E** Immunoblot analysis of CYP7A1. **F** Representative immunohistochemistry images for CYP7A1. **G** Immunohistochemistry analysis of CYP7A1. The scale bar indicates 50 μm. F1, the second generation (offspring); Ctrl, standard diet; HFD, high-fat diet; GO, Gene Ontology; KEGG, Kyoto Encyclopedia of Genes and Genomes; Ldlr, low-density lipoprotein receptor; Sr-bi, scavenger receptor class B type I; Hmgcr, 3 hydroxy-3 methylglutaryl CoA reductase; Cyp7a1, cholesterol 7α-hydroxylase; Cyp8b1, cytochrome P450 family 8 subfamily B member 1; Abcg5, ATP binding cassette subfamily G member 5; Abcg8, ATP binding cassette subfamily G member 8. Data presented as the mean ± SEM. *P < 0.05, **P < 0.01, ***P < 0.001 versus F1-Ctrl group. *n* = 3 litters for each group for RNA-seq. *n* = 6 litters for F1-Ctrl and F1-HFD group, one male offspring per litter

To assess the impact of maternal HFD feeding on the cholesterol metabolism of offspring, we conducted RNA-Seq to investigate the transcriptome of the liver. Differentially expressed genes functionally analyzed using GO revealed changes in the cholesterol metabolic process (Fig. 3A). KEGG pathway analysis revealed the top altered organismal system was related to bile secretion, which plays an important role in cholesterol metabolism (Fig. 3B). It appears that maternal HFD feeding disturbs hepatic cholesterol metabolism in offspring after weaning.

Cholesterol homeostasis involves cholesterol uptake, synthesis, degradation, and efflux. We first examined the expression of cholesterol metabolism genes in mRNA in the livers of the offspring. Maternal HFD feeding increased *Sr-bi* but decreased *Hmgcr* and *Cyp7a1* expression in offspring liver (Fig. 3C). This result indicated HDL cholesterol uptake was increased whereas endogenous cholesterol biosynthesis and cholesterol conversion into bile acids was reduced in offspring liver as a consequence of maternal HFD feeding. Considering the higher hepatic cholesterol content in offspring of HFD-fed dams, liver cholesterol flux from uptake and endogenous biosynthesis exceeds cholesterol degradation and efflux in the offspring. Therefore, CYP7A1 was identified as a key mediator in maternal HFD feeding-induced cholesterol metabolism disturbance in offspring. Consistently, hepatic CYP7A1 protein expression was significantly decreased in offspring from HFD-fed dams, as reflected by both western blot and immunohistochemistry (Fig. 3D–G). These together demonstrate that maternal HFD feeding suppresses CYP7A1 expression, which inhibits cholesterol to bile acid conversion and contributes to increased cholesterol deposition in the liver of the offspring.

### Maternal HFD feeding increases DNA methylation by upregulating DNMT3A in offspring’s liver

**Fig. 4:**
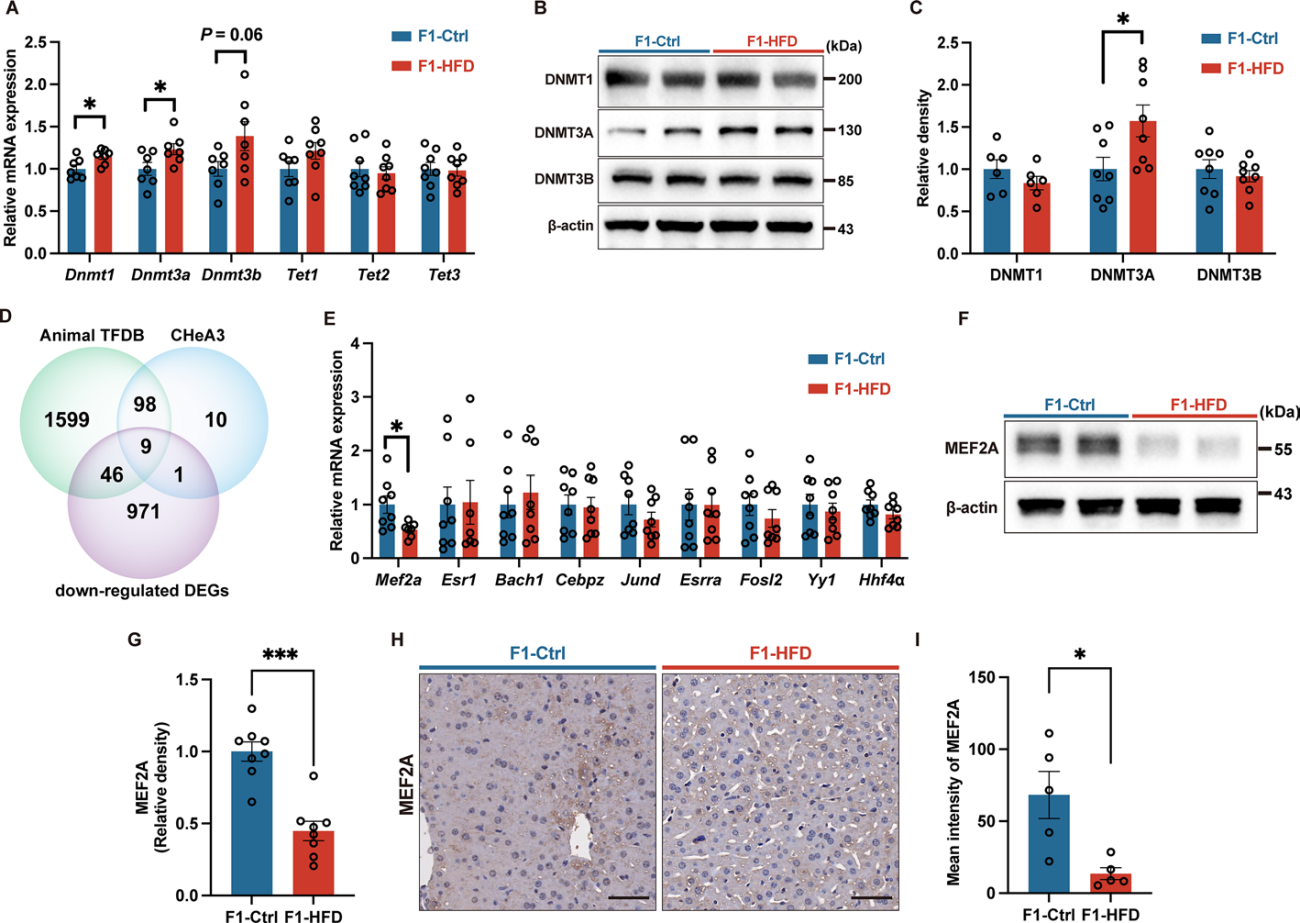
Maternal high-fat feeding upregulates DNMTs expression and downregulates MEF2A transcription factor in offspring.** A** mRNA expression of *Dnmts* and *Tets*. **B** Representative immunoblot images for DNMTs. **C** Immunoblot analysis of DNMT1, DNMT3A, and DNMT3B. **D** Venn diagram of predicted transcription factors and DEGs. **E** mRNA expression of candidate transcription factors. **F** Representative immunoblot images for MEF2A. **G** Immunoblot analysis of MEF2A. **H** Representative immunohistochemistry images for MEF2A. **I** Immunohistochemistry analysis of MEF2A. The scale bar indicates 50 μm. F1, the second generation (offspring); Ctrl, standard diet; HFD, high-fat diet; Dnmts, DNA methyltransferases; Tets, ten-eleven translocation dioxygenases; MEF2A, myocyte enhancer factor 2A; DEGs, differentially expressed genes. Data presented as the mean ± SEM. *P < 0.05, ***P < 0.001 versus F1-Ctrl group. *n* = 5–8 litters for F1-Ctrl group, *n* = 5–8 litters for F1-HFD group, one male offspring per litter

Maternal exposure to an unhealthy diet potentially increases offspring susceptibility to metabolic diseases, which is programmed by epigenetic modifications[28]. As a quintessential epigenetic mechanism, DNA methylation was first investigated by examining the expression of DNA methylation enzymes (DNMTs) and ten-eleven translocation dioxygenases (TETs) demethylation enzymes in offspring liver. Interestingly, the mRNA levels of *Dnmt1* and *Dnmt3a* were increased in the offspring livers from HFD-fed dams, while *Dnmt3b* only showed a trend of increase whereas the expression of *Tet1*, *Tet2*, and *Tet3* remained unaffected (Fig. 4A). Furthermore, we confirmed that DNMT3A protein level was significantly increased in the livers of offspring from HFD-fed dams (Fig. 4B, C). These findings indicated that maternal HFD feeding alters DNA methylation in offspring’s liver at weaning.

### Identification of transcription factor MEF2A that mediates the detrimental effects of maternal HFD on offspring liver cholesterol metabolism

To identify potential regulators of cholesterol metabolism in offspring liver, we analyzed hepatic DEGs between HFD and control groups. Given the elevated DNMT3A protein level in offspring from HFD-fed dams, we hypothesized that maternal HFD feeding might suppress target gene expression through hypermethylation. Therefore, we focused on downregulated genes and predicted possible upstream transcription factors using bioinformatics databases, including the Animal Transcription Factor Database (AnimalTFDB, http://bioinfo.life.hust.edu.cn/AnimalTFDB4/) and ChIP-X Enrichment Analysis 3 (ChEA3, https://maayanlab.cloud/chea3/). Subsequently, based on these databases, we identified nine transcription factors that overlapped and downregulated genes (Fig. 4D), including *Mef2a*, estrogen receptor 1 (*Esr1*), BTB domain and CNC homolog 1 (*Bach1*), CCAAT enhancer binding protein zeta (*Cebpz*), JunD proto-oncogene (*Jund*), estrogen-related receptor alpha (*Esrra*), FOS like 2, AP-1 transcription factor subunit (*Fosl2*), YY1 transcription factor (*Yy1*), and hepatocyte nuclear factor 4α (*Hnf4α*) (Fig. 4E). Among the identified transcription factors, only *Mef2a* showed significantly decreased mRNA expression in the liver of offspring due to maternal HFD feeding (Fig. 4E). Furthermore, protein expression analysis confirmed the decrease in MEF2A levels in the liver of offspring from HFD-fed dams via both western blot and immunohistochemistry (Fig. 4F–I). Therefore, MEF2A may be the key transcription factor mediating the detrimental effects of maternal HFD feeding on cholesterol metabolism in offspring.

### MEF2A regulates cholesterol metabolism through CYP7A1

**Fig. 5:**
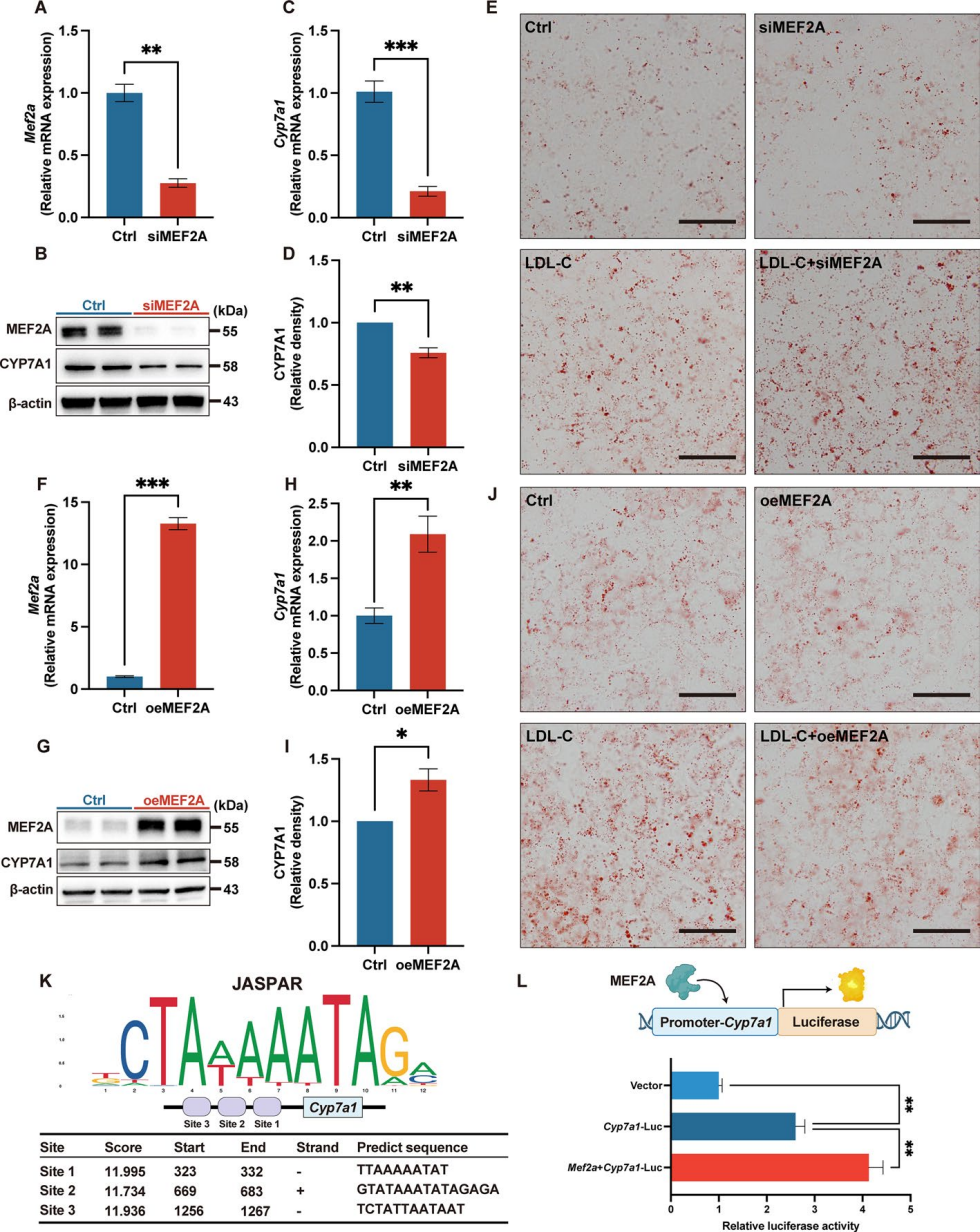
MEF2A regulates cholesterol metabolism via targeting CYP7A1.** A** mRNA expression of *Mef2a*. **B** Representative immunoblot images for MEF2A and CYP7A1. **C** mRNA expression of *Cyp7a1*. **D** Immunoblot analysis of CYP7A1. **E** Representative images of Oil Red O staining of HepG2 cells. **F** mRNA expression of *Mef2a*. **G**; Representative immunoblot images for MEF2A and CYP7A1. **H** mRNA expression of *Cyp7a1*. **I** Immunoblot analysis of CYP7A1. **J** Representative images of Oil Red O staining of HepG2 cells. **K** Predicted MEF2A transcription factor binding sites in Cyp7a1 promoter. **L** Relative luciferase activity of dual luciferase assay. The scale bar indicates 50 μm. Ctrl, control; siMEF2A, down-regulation of MEF2A; oeMEF2A, over-expression of MEF2A; MEF2A, myocyte enhancer factor 2A; CYP7A1, cholesterol 7α-hydroxylase. Data presented as the mean ± SEM. *P < 0.05, **P < 0.01, ***P < 0.001 versus Ctrl *n* = 3–5

To examine how MEF2A affects cholesterol metabolism, we employed *Mef2a* siRNA (siMEF2A) in HepG2 cells to reduce MEF2A expression. After treatment, both MEF2A mRNA and protein levels displayed a 70–80% reduction in *Mef2a* siRNA-treated group compared with the control (Fig. 5A, B). Moreover, MEF2A knockdown significantly decreased the mRNA and protein expression of CYP7A1 (Fig. 5B–D). Consistently, Oil Red O staining suggested that the knockdown of MEF2A exacerbated lipid accumulation in LDL-cholesterol-treated HepG2 cells (Fig. 5E). In contrast, lentivirus-induced overexpression of MEF2A increased the expression of Cyp7a1 gene and protein levels in HepG2 cells (Fig. 5F–I). Accordingly, the lipid accumulation was also decreased in LDL-C-treated MEF2A-overexpressed HepG2 cells (Fig. 5J). Collectively, these findings suggest that knockdown of MEF2A can suppress CYP7A1 expression and exacerbate lipid accumulation in HepG2 cells.

Furthermore, to validate whether MEF2A regulates the transcription of *Cyp7a1,*, the potential MEF2A binding sites on Cyp7a1 promoter were predicted using the JASPAR database (https://jaspar.elixir.no). It showed that MEF2A can bind to three promoter sequences of Cyp7a1 with relatively high scores (Fig. 5K). We further generated a *Cyp7a1* promoter-luciferase reporter construct and performed dual luciferase reporter assays. MEF2A greatly promoted *Cyp7a1* transcription (Fig. 5L). Collectively, these findings elucidate the critical role of MEF2A in controlling cholesterol metabolism by directly regulating *Cyp7a1* transcription.

### Maternal HFD feeding induces hepatic *Mef2a* hypermethylation in offspring during the fetal period

**Fig. 6:**
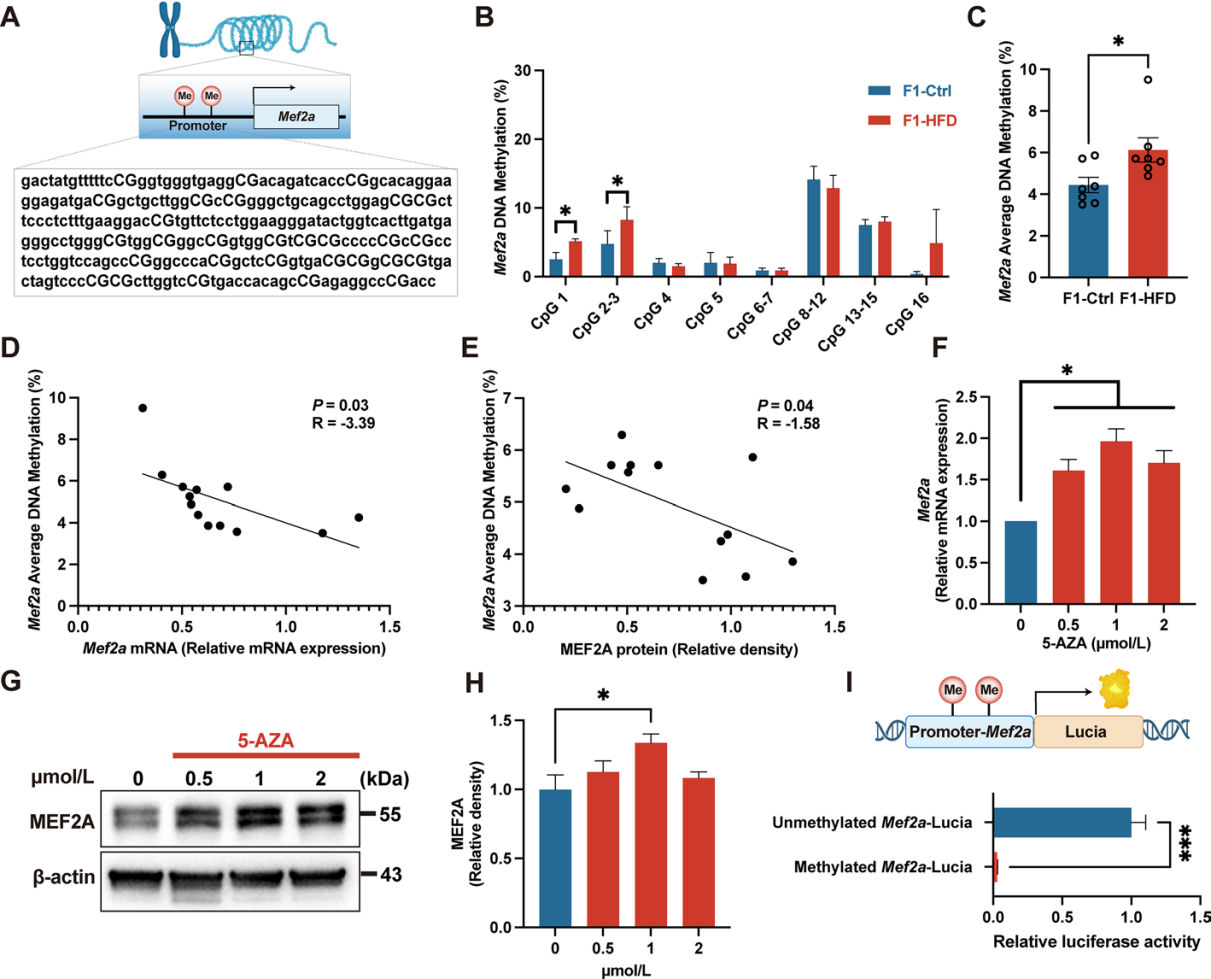
Maternal high-fat diet feeding induces hepatic *mef2a* hypermethylation in offspring. **A** CpG sites measured in *Mef2a* promoter. **B** DNA methylation levels of different CpG sites in *Mef2a* promoter. **C** Average DNA methylation level in *Mef2a* promoter. **D** Correlation between *Mef2a* methylation level and *Mef2a* mRNA expression.** E** Correlation between *Mef2a* methylation level and MEF2A protein expression. **F** mRNA expression of *Mef2a* in 5-AZA-treated HepG2 cells. **G** Representative immunoblot images for MEF2A in 5-AZA-treated HepG2 cells. **H** Immunoblot analysis of MEF2A in 5-AZA-treated HepG2cells. **I** Relative luciferase activity of methylated or unmethylated plasmids in H9C2 cells. F1, the second generation (offspring); Ctrl, standard diet; HFD, high-fat diet; MEF2A, myocyte enhancer factor 2A; 5-AZA, 5-azacitidine. Data presented as the mean ± SEM. *P < 0.05, ***P < 0.001 versus F1-Ctrl group. *n* = 7–8 litters/group, one male offspring per litter. *P < 0.05, ***P < 0.001 versus Ctrl. *n* = 6–7 litters for F1-Ctrl group, *n* = 6–7 litters for F1-HFD group, one male offspring per litter

To further investigate whether the diminished expression of *Mef2a* results from DNA hypermethylation, we examined the CpG sites in the promoter region of *Mef2a* and identified a CpG island (Fig. 6A). Methylation levels at CpG sites 1–3, as well as the average DNA methylation levels, were markedly elevated in the livers of offspring of HFD dams compared with those of control dams. (Fig. 6B, C). Moreover, a negative correlation was observed between the average DNA methylation of *Mef2a* and the expression of MEF2A (Fig. 6D, E). To ascertain whether MEF2A expression is influenced by DNA methylation, HepG2 cells were treated with 5-AZA, a DNA demethylating agent. 5-AZA exposure significantly increased MEF2A expression on both mRNA and protein levels. (Fig. 6F–H). Furthermore, to evaluate the impact of DNA methylation on *Mef2a* expression, luciferase reporter constructs containing the human *Mef2a* promoter with or without CpG dinucleotides were generated. H9C2 cells are readily transfectable and exhibit higher expression levels of MEF2A. Thus, to evaluate the impact of DNA methylation on *Mef2a* expression, we conducted this experiment in H9C2 cells. It showed that the *Mef2a* promoter activity was notably suppressed in the presence of CpG dinucleotides compared with the mutant in H9C2 cells (Fig. 6I), indicating that DNA methylation at the *Mef2a* promoter indeed regulates the expression of *Mef2a*.

**Fig. 7:**
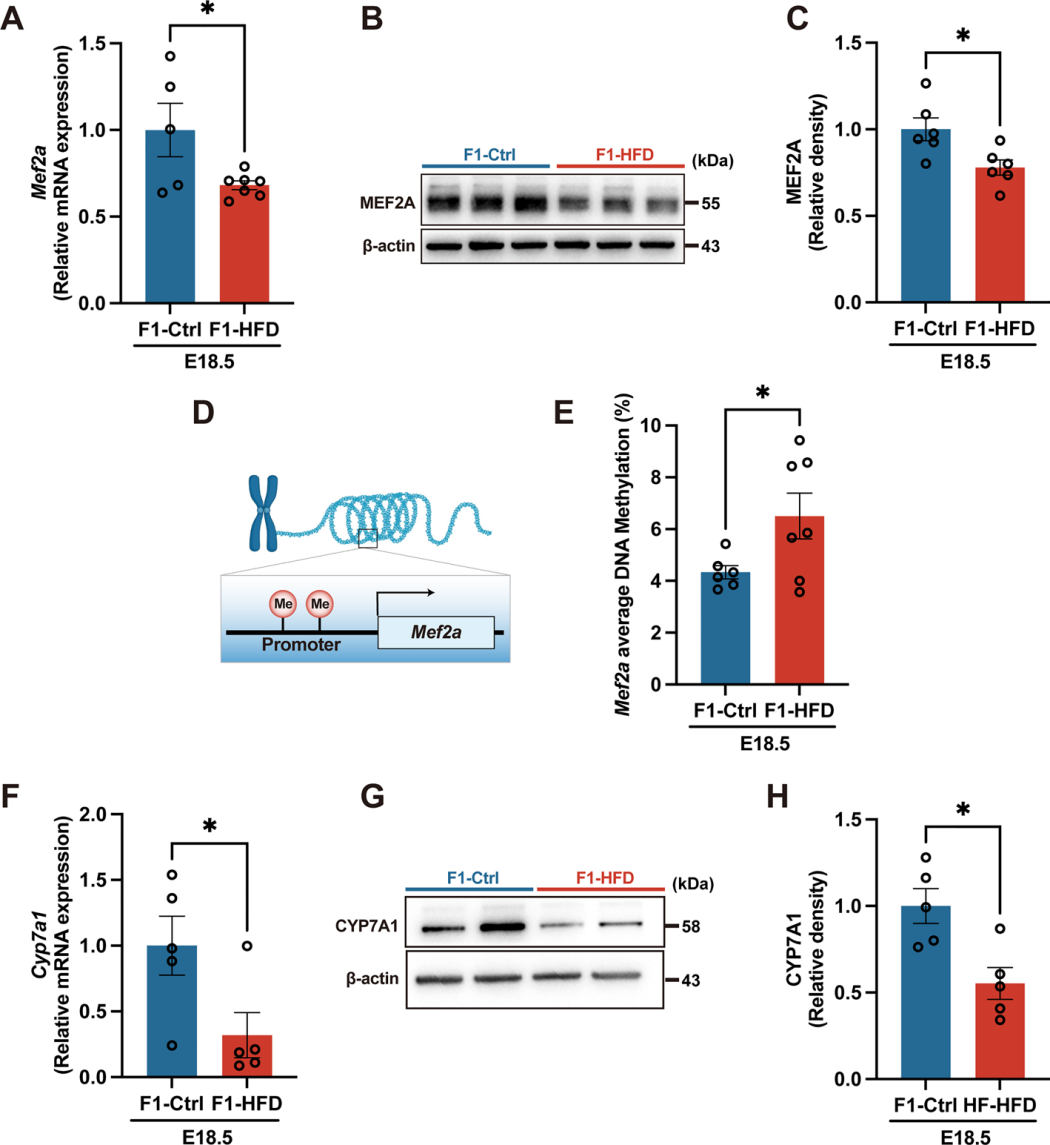
Maternal high-fat diet feeding contributes to hepatic *Mef2a* hypermethylation in fetus. **A** mRNA expression of *Mef2a* in fetal liver. **B** Representative immunoblot images for MEF2A in fetal liver. **C** Immunoblot analysis of MEF2A in fetal liver. **D,E** Average DNA methylation level in *Mef2a* promoter. **F–H** CYP7A1 mRNA and protein expression in the fetal liver. F1, the second generation (offspring); Ctrl, standard diet; HFD, high-fat diet; E18.5, embryonic day 18.5; MEF2A, myocyte enhancer factor 2A; CYP7A1, cholesterol 7α-hydroxylase. Data presented as the mean ± SEM. *P < 0.05 versus F1-Ctrl. *n* = 5–6 litters for F1-Ctrl group, *n* = 5–7 litters for F1-HFD group, one male offspring per litter

To further investigate when DNA hypermethylation occurs in the offspring liver, we also analyzed the E18.5 fetus liver samples. Both mRNA and protein expression of MEF2A were diminished in the fetal livers from HFD dams compared with controls (Fig. 7A–C). Additionally, the average DNA methylation level of *Mef2a* was consistently higher in the fetal livers from HFD dams (Fig. 7D, E). Accordingly, CYP7A1 expression was significantly decreased in the fetal liver of dams fed with HFD diet (Fig. 7F–H). On the basis of these findings, maternal HFD feeding-induced hypermethylation of *Mef2a* in offspring may occur as early as the fetal period.

## Discussion

**Fig. 8:**
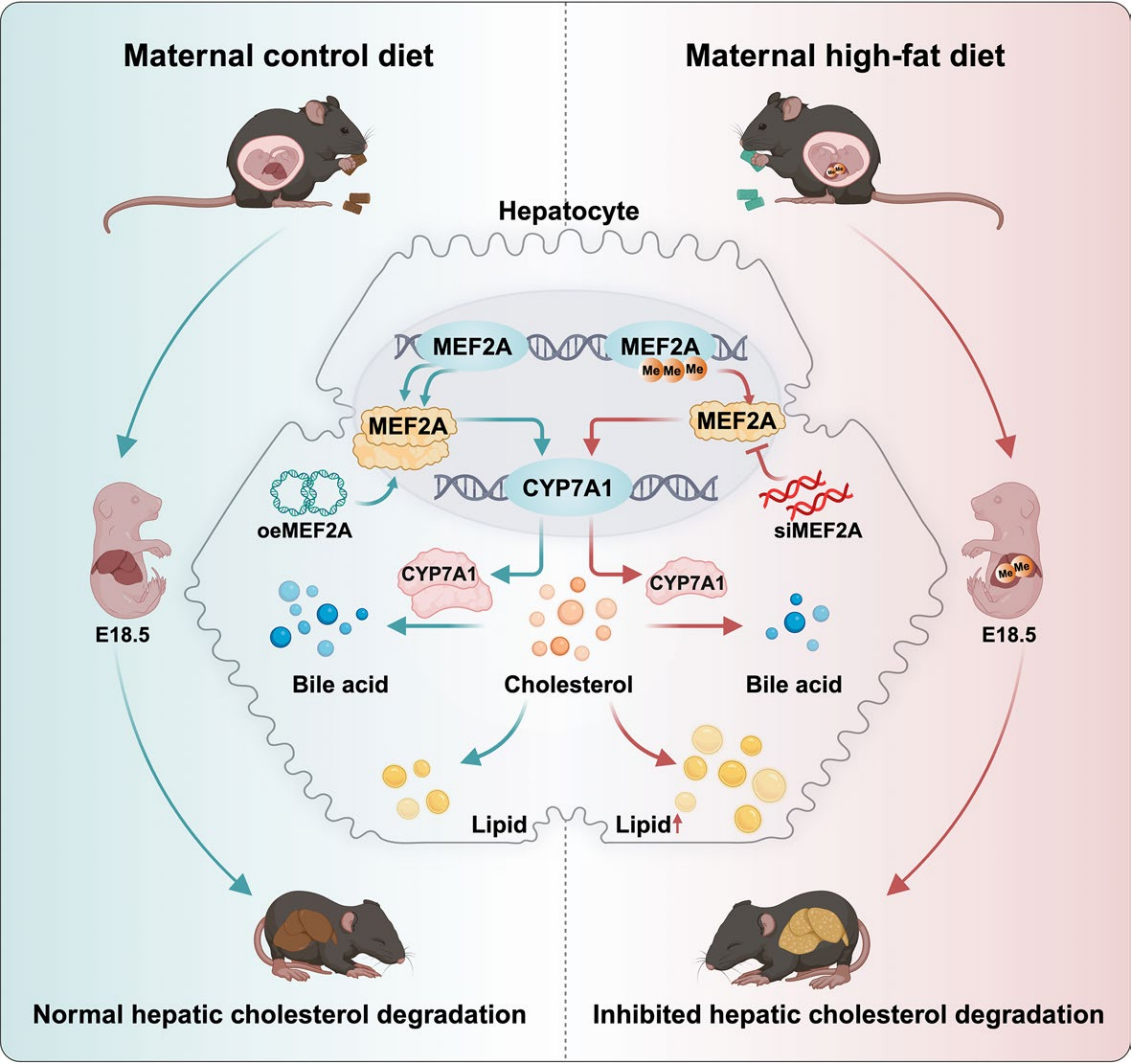
Summary figure. Schematic diagram illustrating the mechanisms of maternal high-fat diet feeding in hepatic lipid accumulation in offspring mice in the study. Maternal high-fat diet can mediate DNA methylation changes in *Mef2a*, leading to suppression of hepatic CYP7A1 expression and subsequent hepatic cholesterol accumulation in offspring at weaning age. MEF2A, myocyte enhancer factor 2A; CYP7A1, cholesterol 7α-hydroxylase

Diabetes and obesity have become more prevalent among women of reproductive age [29], profoundly impacting the metabolic health of future generations. The detrimental metabolic effects of maternal HFD can be long-lasting for offspring [14, 15]. As a key epigenetic mechanism, DNA methylation mediates the impact of a mother’s unhealthy diet on the metabolism of offspring [13–15]. However, its specific influence on offspring cholesterol metabolism remains poorly understood. Our study indicates that maternal HFD can mediate DNA methylation changes in *Mef2a*, leading to suppression of hepatic CYP7A1 expression, which inhibits cholesterol elimination to bile acids, and subsequently induces hepatic cholesterol accumulation in offspring as early as weaning age (Fig. 8).

In this study, we investigated how maternal high-fat feeding affects offspring cholesterol metabolism and the underlying mechanisms involved. We observed that maternal HFD contributed to increased body weight, hypercholesteremia, and hepatic lipid accumulation in offspring. This was accompanied by repression of hepatic CYP7A1 expression, resulting in cholesterol in the liver of offspring from HFD-fed dams. Cholesterol homeostasis, essential for optimal cellular and organismal function, has an intrauterine developmental origin [16]. While previous research has extensively explored the programming effects of maternal overnutrition on disturbed glucose balance in offspring [13, 30], studies about the developmental origin of cholesterol metabolism are limited.

Consistent with previous research [12], our results showed that maternal HFD consumption resulted in elevated serum T-CHO, LDL-C concentration, as well as hepatic cholesterol accumulation in offspring. CYP7A1 deficiency has been linked to hypercholesteremia [31] and atherosclerosis [32], while its overexpression reduced cholesterol levels both in serum and liver [33, 34]. We also observed elevated expression of *Sr-bi*, indicating increased uptake of HDL-C. However, the low expression of *Hmgcr* may be attributed to the suppressive effect of high intracellular cholesterol levels on the endogenous cholesterol biosynthesis pathway. Given the higher hepatic cholesterol content observed in the offspring of HFD-fed dams, it appears that the influx of cholesterol from dietary intake and endogenous biosynthesis exceeds its degradation and efflux. Therefore, we propose that the detrimental effects of maternal high-fat feeding on hepatic cholesterol metabolism in offspring are primarily attributable to the suppression of CYP7A1 expression.

We further identified MEF2A as an important transcription factor in the offspring liver that can directly modulate CYP7A1 expression. MEF2A, involved in cellular processes including proliferation, differentiation, and survival, has been implicated in glucose and lipid metabolism [35]. However, studies documenting the role of MEF2A in cholesterol metabolism is limited. Research in *Drosophila* has shown that muscle-specific attenuation of MEF2 contributes to the accumulation of large intramuscular lipid droplets enriched in cholesterol esters when exposed to high-calorie carbohydrate-sufficient diets [36]. Our study demonstrates that MEF2A governs cholesterol metabolism by directly modulating CYP7A1 expression in vitro, underscoring its involvement in maintaining cholesterol homeostasis.

This is consistent with numerous studies establishing DNA methylation as a critical mediator linking maternal adverse nutrition to metabolic disturbances in offspring [14, 15]. DNA methylation is mediated by DNMT enzymes, and DNA demethylation is mediated by TET enzymes [37]. The insufficiency of TET3 induced by maternal hyperglycemia conferred a predisposition to glucose intolerance in the next generation through hypermethylation of the glucokinase gene in pancreatic islets [38]. DNMT1 inhibition ameliorated cholesterol accumulation through Dnmt1-dependent epigenetic mechanism in mice [39]. As compared with nonobese humans, a higher expression of DNMT1 and DNMT3b was observed in obese humans [40]. However, the role of DNMTs in mediating the impact of a mother’s health on the offspring’s metabolism is less well documented. Our study revealed that alterations in DNMTs, rather than TETs, were observed in the liver of offspring, leading to the hypermethylation of *Mef2a*. In addition, DNA hypermethylation in the *Mef2a* promoter region resulted in hepatic cholesterol accumulation in offspring through suppressing CYP7A1. We further found that epigenetic modifications may manifest as early as the fetal stage, with increased DNA methylation levels on *Mef2a* in fetal liver samples.

In addition, since no significant difference in body weight was observed between dams fed with a standard diet and a high-fat diet prior to breeding, during gestation, and lactation, our study highlighted the importance of the high fat diet consumption during critical windows of programming. Both pregnancy and lactation are important, even if body weight was not increased. Thus, our study emphasizes that it may be unnecessary to increase body weight to programming key cholesterol pathways and hypermethylation, and that the changes observed are independent of the weight gained prior to gestation, during gestation, and lactation. Thus, it is important to mention that only high-fat diet intake during this period can adversely program offspring.

Our study has several strengths, including its novel demonstration of maternal HFD’s effects on offspring cholesterol metabolism, identification of MEF2A as a key transcription factor, and elucidation of *Mef2a* methylation’s role in transgenerational effects. However, limitations include the use of only HepG2 cells for in vitro studies, and challenges in gene editing for in vivo transgenerational mouse models.

## Conclusions

Our findings suggest that lipid metabolic disturbances in offspring induced by maternal HFD are mediated by DNA methylation changes in MEF2A, leading to suppression of hepatic CYP7A1 expression and subsequent cholesterol accumulation in the liver by weaning age. Moreover, DNA hypermethylation of MEF2A may occur as early as the fetal period. By elucidating these epigenetic mechanisms, our study underscores the critical importance of maternal overnutrition in shaping offspring health outcomes from an early developmental stage.

## Data Availability

All data relevant to the study are included in the article or uploaded as supplementary information.

## Abbreviations

AnimalTFDB: Animal transcription factor database
BACH1: BTB domain and CNC homolog 1
CEBPZ: CCAAT enhancer binding protein zeta
ChEA3: ChIP-X enrichment analysis 3
CYP7A1: Cholesterol 7-alpha hydroxylase
DAB: Diaminobenzidine
DEG: Differentially expressed genes
Dio3os: Dio3 antisense RNA
DNMT: DNA methyltransferases
ECL: Enhanced chemiluminescence
ESR1: Estrogen receptor 1
ESRRA: Estrogen-related receptor alpha
FBS: Fetal bovine serum
FOSL2: FOS like 2, AP-1 transcription factor subunit
GO: Gene ontology
H&E: Hematoxylin–eosin
HepG2: Human hepatocellular carcinoma cell lines
HFD: High-fat diet
HNF4α: Hepatocyte nuclear factor 4α
JUND: JunD proto-oncogene
KEGG: Kyoto Encyclopedia of Genes and Genomes
LDL-C: Low-density lipoprotein cholesterol
MEF2A: Myocyte enhancer factor 2 A
PPARα: Peroxisome proliferator-activated receptor-α
T-CHO: Total cholesterol
TET: Ten-eleven translocation (TET) dioxygenases
TG: Triglyceride
YY1: YY1 transcription factor
5-AZA: Azacitidine
